# Gut microbiota and microbiota-based therapies for *Clostridioides difficile* infection

**DOI:** 10.3389/fmed.2022.1093329

**Published:** 2023-01-09

**Authors:** Teena Chopra, Gail Hecht, Glenn Tillotson

**Affiliations:** ^1^Division of Infectious Diseases, Wayne State University, Detroit, MI, United States; ^2^Department of Medicine, Loyola University Chicago Stritch School of Medicine, Maywood, IL, United States; ^3^GST Micro LLC, Henrico, VA, United States

**Keywords:** microbiota, microbiome, fecal microbiota transplant, *Clostridioides difficile* infection, *Clostridium difficile*, recurrent CDI

## Abstract

*Clostridioides difficile* infection poses significant clinical challenges due to its recurrent nature. Current antibiotic management does not address the underlying issue, that of a disturbed gastrointestinal microbiome, called dysbiosis. This provides a supportive environment for the germination of *C. difficile* spores which lead to infection and toxin production as well as an array of other health conditions. The use of microbiome restoration therapies such as live biotherapeutics can reverse dysbiosis and lead to good clinical outcomes. Several such therapies are under clinical investigation.

## 1. Introduction

*Clostridioides difficile* infection (CDI) is an urgent threat, both for the patient and healthcare professionals. CDI is one of the most common healthcare-associated infections and aggressive action is required to combat this threat (1). There are an estimated 467,400 cases of healthcare- and community-associated CDI cases annually in the United States and a cumulative incidence of 8 per 100,000 person-years in the European Union (2, 3). The estimated direct medical cost of CDI in the US is $5.4 billion (2014 dollars) (4).

Patients with CDI often present with watery diarrhea and abdominal pain, but symptoms can also include fever, hypotension, or ileus in more severe cases (5); complications can include sepsis or colectomy/ileostomy (6–11). Testing for CDI is recommended for patients who have unexplained, new onset diarrhea (at least 3 unformed stools over ≥24 h) using a nucleic acid amplification test alone or as part of an algorithm that includes glutamate dehydrogenase or stool toxin test (12). The current recommended treatment regimen for an initial episode of CDI is fidaxomicin (200 mg BID q10d) or vancomycin (125 mg, QID q10d) as an acceptable alternative (13).

Unfortunately, in approximately 25% of cases, CDI recurs within 1–2 months of the initial infection (6, 7, 14, 15). Recurrence is often associated with more severe disease, increased costs, and hypervirulent strains of *C. difficile* (16–19). After a first recurrence, patients are substantially more likely to have a subsequent recurrence, with approximately 50–60% of these patients experiencing multiply recurrent CDI (6, 7, 20, 21).

## 2. Gut dysbiosis and *Clostridioides difficile* infection

Initial episodes of CDI are almost always precipitated by antibiotic use, so much so that it has the strongest association of any identified risk factor for CDI (6, 7, 22, 23). Other common risk factors for CDI include older age, use of gastric acid suppressants, comorbid conditions such as kidney disease and cardiovascular disease, and recent healthcare exposure (24–27).

*Clostridioides difficile* is found in the gut of some healthy individuals and is kept in check, residing in a dormant spore state, by a healthy gut microbiota (28). Underlying the pathophysiology of CDI is disruption of the gut microbiota, or gut dysbiosis. Dysbiosis has been defined as “any change to the composition of resident commensal communities relative to [those] found in healthy individuals” (29). This can include a loss of beneficial microbes, reduced diversity of gut species, or expansion of a pathogenic species (29). In patients with CDI, the gut microbiota exhibits a loss of diversity, which can worsen with recurrent CDI (30). With gut dysbiosis, *C. difficile* spores can germinate and produce exotoxins, disrupting the intestinal mucosa and causing CDI-associated diarrhea (31, 32).

The inciting dysbiosis for CDI can arise for several reasons. Antibiotics that are considered significant disruptors of the gut microbiota also have the strongest association with developing CDI (33–37). Older age brings changes in the gut microbiota, which could be influenced by a change in diet, lifestyle, or immune senescence (30, 38, 39). Patients taking chronic gastric acid suppressants, who are often older, show significant increases in gut *Enterococcus*, *Streptococcus*, and *Staphylococcus* species (40).

*Clostridioides difficile* spores require a germinant to transform from the spore state to the growing, vegetative cell, in the form of specific bile acids. Primary bile acids are synthesized by hepatocytes and transformed into secondary bile acids by certain members of the healthy gut microbiota (28, 41). Bile acids derived from cholic acid promote the germination of *C. difficile* spores, while bile acids derived from chenodeoxycholic acid (CDCA) inhibit germination (41). In addition, vegetative cell growth of *C. difficile* is inhibited by CDCA. In animal studies and in humans, hosts with higher levels of secondary bile acids were more resistant to developing CDI, whereas hosts with higher levels of primary bile acids were more susceptible (41).

Perhaps counterintuitively, CDI is treated with antibiotics. While antibiotics may eliminate the initial infection, they alter the composition of the gut microbiota, including widespread reduction in diversity by commonly-used vancomycin (29, 30). With the continued burden of recurrent CDI, that does not appear to be lessening with increased infection control measures or changes in antimicrobial prescribing, a non-antibiotic approach may offer an alternative means of addressing the disease (2).

## 3. Restoring the gut microbiota in *Clostridioides difficile* infection

Given the underlying state of gut dysbiosis that fosters CDI, an ideal goal for patients with CDI is eubiosis, or restoring the gut microbiota to a healthy state (28, 29). Microbiota-based therapies have been investigated by Western medicine as a treatment for gut dysbiosis since the 1950s (42). Since then, their use has increased steadily, in parallel with our understanding of gut microbiota disruption as an underlying cause of CDI as well as many other gastrointestinal disorders.

Fecal microbiota transplantation (FMT) is the delivery of intestinal microbiota from a healthy donor to a recipient to mitigate disease by modifying the structure and/or function of the gut microbiota (43, 44). FMT is currently recommended in the CDI treatment guidelines as an option at the second or subsequent recurrence (12, 45). In addition to CDI treatment, including FMT, changes to underlying risk factors should be considered for their effect on the gut microbiota, such as discontinuing gastric acid suppressants or altering systemic antimicrobial therapy for a non-CDI infection.

The goal of FMT is to restore the gut microbiota to a healthy state and replace dysbiotic microbes with taxa/species that are associated with healthy host microbiota (46, 47). The expectation is that reintroduced healthy species will engraft and out-compete *C. difficile*, thus eliminating dysbiosis and providing colonization resistance (48). FMT can return metabolite levels and profiles, including bile acids and short-chain fatty acids, to a healthy state, presumably as a result of enzymatic activity provided by normal host microbiota (48).

Reduced presence of *Bacteroides* spp. appears to be associated with negative consequences for GI disorders, including CDI (49). Bacteria in the phyla Bacteroidetes are abundant in healthy gut microbiota and likely play a key role in bacterial metabolism and the gut environment (28). The presence of *Bacteroides* spp. and their surface proteins and metabolites may activate the host immune system to limit entry and proliferation of potential pathogens or exert an antibacterial effect (50, 51).

The initial literature regarding FMT for CDI was primarily case reports and retrospective cohort studies as the therapy was being investigated (52–54). While these studies often showed positive patient outcomes, namely prevention of CDI recurrence for several months after treatment, by nature of their study design the resulting data were prone to selection bias. More recently, prospective and randomized controlled trials of FMT have been completed, generally demonstrating FMT as a safe and effective therapy for CDI with treatment success rates of ∼75% (55–57). A recent prospective, real-world observational study of medically complex patients receiving FMT for CDI reported 78% (4,195/5,344) of patients exhibited clinical cure, with 3.6% of patients experiencing a serious adverse event (58). FMT has also been shown to decrease mortality in patients with refractory severe or fulminant CDI (59).

Performing FMT can be operationally challenging, including costs and logistical concerns around screening donors and processing stool (58, 60). Additionally, there is no standard protocol for FMT composition, route of delivery, number of infusions, or dosage, variables that could all affect treatment outcomes (61).

## 4. Approaches to restoring the gut microbiota

Live biotherapeutic products (LBPs) have been developed as an extension of the initial FMT studies, in part as a way to standardize products and outcomes being measured. LBPs contain live microbes that are able to prevent, treat or cure a disease (62). Several LBPs have been or are currently being studied for CDI. The goal of treatment with LBPs for CDI is similar to FMT, namely to restore the gut microbiota to a healthier state (63).

LBPs that are currently in late-stage development differ in their approach toward product composition and delivery. SER-109 (Seres Pharmaceuticals, Lexington MA) is an oral capsule (4 capsules once daily q3d) containing spores of ∼50 specific species of only Firmicutes that are isolated from healthy donors (64). SER-109 was designed on the premise that Firmicutes can compete metabolically with *C. difficile* for essential nutrients and bile acids (63). While a phase 2 study of SER-109 did not show a significant difference versus placebo in patients with multiply recurrent CDI, in those patients who did show SER-109 engraftment by microbiome analysis, there was also a significant increase in secondary bile acids (65). From a phase 3 study of patients who had 3 or more episodes of CDI, the treatment success after SER-109 was 88% (recurrence rate of 12%) (66).

RBX2660 (Ferring Pharmaceuticals, Parsippany NJ) is a biologically-sourced, broad consortium microbiota-based live biotherapeutic product (LBP) that is processed from the stool of healthy donors, standardized and administered rectally (67). The product was approved on November 30, 2022 by the FDA as Rebyota as a live biotherapeutic for the treatment of recurrent *C. difficile* infection (REBYOTA | FDA) RBX2660 is screened for 29 different species of pathogens as shown in Table 1. Results from a phase 3 trial of RBX2660, analyzed with a Bayesian hierarchical model formally incorporating data from a phase 2b trial, showed a treatment success rate of 70.6% (68). Long-term data (up to 24 months) after treatment with RBX2660 in a phase 2 trial showed durable treatment success, with more than 90% of treatment responders remaining CDI-free at 6, 12, and 24 months (69, 70). Microbiota analyses from this phase 2 trial also showed a highly dysbiotic composition before treatment, which converged toward the RBX2660 composition within 7 days after treatment (69, 71). Taxa that were restored to predominance after RBX2660 included Bacteroidia and Clostridia while gammaproteobacteria and bacilli, the deleterious organisms, were reduced. Administration of RBX2660 delivery is via enema, without the need for bowel preparation or colonoscopy and can be used in patients who are not able to take an oral product.

**TABLE 1:** Pathogen screening on RBX2660.

Pathogens		Multi-drug resistant organisms
*Clostridioides difficile* A/B	*Plesiomonas shigelloides*	Extended spectrum beta-lactamase (ESBL)
Enteroaggregative *E. coli* (EAEC)	*Campylobacte*r species	Vancomycin-resistant Enterococci (VRE)
Enterotoxigenic *E. coli* (ETEC)	*Salmonella* species	Carbapenem-resistant Enterobacterales
Enteropathogenic *E. coli* (EPEC)	Vibrio species/cholerae	Methicillin-resistant *Staphylococcus aureus* (MRSA)
Entamoeba histolytica	*Yersinia enterocolitica*	
Astrovirus	Shiga-like-toxin-producer *E. coli* (STEC) Shigella/Enteroinvasive *E. coli* (EIEC)	
Sapovirus (Genogroups I, II, IV, V)	*Giardia lamblia*	
Listeria culture	Norovirus GI/GII	
Cryptosporidium	Rotavirus A	
Cyclospora	Adenovirus F40/41	
Cystoisospora		
Ova and Parasite exam		
Aeromonas		

CP101 (Finch Therapeutics, Somerville, MA, USA) is an oral capsule (10 capsules taken once) delivering a full-spectrum microbiota product that showed 75% efficacy in preventing CDI recurrence in a phase 2 trial (72). A phase 3 trial of CP101 is currently recruiting patients (NCT05153499). Several other microbiota-based products in earlier stages of development have been or are currently being investigated for CDI (63).

The negative physical effects of gut dysbiosis are clear, but emerging evidence also points to psychological effects as well. Psychological consequences of CDI are reported by ∼70% of people who have active or previous infection (73). From an analysis of Medicare Fee-for-service beneficiaries, within a 12-month period after an initial CDI episode, approximately 15–20% of the cohort had newly diagnosed psychiatric conditions (anxiety, depression, delirium) (7). After receiving a microbiota-based LBP for CDI treatment in a phase 2 trial setting, participants exhibited statistically significant and clinically meaningful improvements in the mental component score of the SF-36 assessment of quality of life (QoL) (74). From a phase 3 randomized, controlled trial, using a the CDiff32, a CDI-specific measure of QoL, patients receiving an LBP reported significant improvements in mental health-related QoL as early as week 1, which continued throughout the 8-week blinded study period (75). While definitive mechanisms linking changes in the gut microbiota to mental state have not been determined, it is clear that there is a link (76).

## 5. Discussion

A healthy gut microbiota is associated with many aspects of health and resistance to CDI as well as other diseases. Restoring healthy gut microbial communities can help break the vicious cycle of recurrence in CDI patients. The outcomes of treatment with live biotherapeutic products have been measured in terms of short- and long-term clinical observations and microbiome changes, which modify the metabolic processes in the gut and elicit positive changes in mental aspects associated with CDI. The availability of regulated standardized products will be welcome additions to the armamentarium against *C. difficile* infections.

## Author contributions

TC and GT conceived the idea for the work, wrote the original draft, revised and edited the manuscript. GH wrote the original draft and revised and edited the manuscript. All authors agreed to be accountable for the content of the work and approved the final version for publication.
